# Feasibility of teleyoga for people with post COVID-19 condition– a mixed method design

**DOI:** 10.1186/s12906-024-04735-4

**Published:** 2025-01-08

**Authors:** Marie Lundberg, Leonie Klompstra, Lotti Orwelius, Mirjam Schimanke, Cecilia Olsson, Anna Strömberg

**Affiliations:** 1 Department of Health Medicine and Caring Sciences, Linkoping University, Linköping, Sweden; 2 Department of Intensive Care, Linköping University, Linköping, Sweden; 3 Department of Biomedical and Clinical Sciences, Linkoping University, Linköping, Sweden; 4 Department of Internal Medicine, Vrinnevi Hospital, Norrköping, Sweden; 5 Department of Health Sciences, Karlstad University, Karlstad, Sweden; 6 Department of Bachelor’s in Nursing, Lovisenberg Diaconal University College, Oslo, Norway; 7 Department of Cardiology, Linkoping University, Linköping, Sweden

**Keywords:** Post covid-19 condition, Rehabilitation, Teleyoga, Yoga, Feasibility study, Mixed method, Mobile application, Videoconference

## Abstract

**Background:**

Evidence about rehabilitation of post COVID-19 condition is scarce. Yoga has been found beneficial in other chronic conditions and can be delivered in a digital format at home. The aim of the study was to explore the feasibility of teleyoga in persons with post COVID-19 condition by assessing adherence, safety, limited efficacy and experiences.

**Methods:**

Pre-post mixed-method design. Participants were recruited from a post COVID-19 rehabilitation clinic. The intervention included standardised live-streamed teleyoga sessions twice/week for 7 weeks and individual yoga using a digital application during 12 weeks. Adherence to the teleyoga intervention was measured by registration of participation and by analysing the log in the application. Safety was measured by registration of serious adverse events. Limited efficacy examined trends in the predicted direction for better outcome in patients with a post-COVID condition in the 6-minute walk test, gait speed, cognition, health-related quality-of-life, mental distress, sleep and exercise motivation. We also assessed patients’ experiences after the intervention.

**Results:**

Nine women and 2 men were enrolled, aged between 27 and 61 years, and duration of post COVID-19 3–12 months. Adherence: Half of the participants participated in more than 50% of the online yoga sessions. They enjoyed the digital format and the social aspect of the online yoga sessions. Some participants experienced that the yoga sessions of 60 min were too long. People with post COVID-19 felt motivated to participate, however they felt conflicted when other commitments took time away from yoga. Adherence to the yoga application varied, 6 patients used it less than 50% of the recommended time. Safety: Persons with post COVID-19 experienced symptoms due to their disease, which could increase during the yoga sessions that made it harder to participate. There were no reported serious adverse events. Limited efficacy: Participants expressed that they breathed more consciously and experienced relaxation and reduction of stress and anxiety. After 7 weeks of teleyoga there was a significant improvement in cognitive function (p-value = 0.048). No differences were found in the physical tests, health-related quality-of-life, anxiety and depression, sleep or in exercise motivation.

**Conclusion:**

Adherence to the online yoga sessions was quite low and might be improved with shorter yoga sessions. Online yoga was safe, but some participants experienced an increase in symptoms. Teleyoga was associated with improved cognition, breathing and relaxation. The results show that online yoga could be feasible for people post COVID-19, but adaptation of the yoga-program may be required, especially as many patients experience an increase of symptoms. Furthermore, the teleyoga should be more flexible with regards to the duration and the number of sessions. As few participants were adherent to the application, the relevance and usefulness of this needs to be further explored.

**Supplementary Information:**

The online version contains supplementary material available at 10.1186/s12906-024-04735-4.

## Background

COVID-19 has killed millions of people and infected over a half billion people worldwide [1, 2]. Most people infected recover after a few days to a few weeks, but some have long-term symptoms. Persisting, atypical and/or new symptoms lasting longer than 12 weeks after a COVID-19 infection are called different names like post-COVID syndrome or long-haulers [3]. In this article we use WHO’s terminology: post COVID-19 condition [4]. Between 10 and 20% of persons with post COVID- 19 condition experience complications due to the disease more than 12 weeks post-infection [5]. It is defined as the continuation or development of new symptoms 3 months after the initial SARS-CoV-2 infection, with these symptoms lasting for at least 2 months with no other explanation [4]. 

The post COVID-19 diagnosis is based on persisting symptoms like dyspnea, cough, racing heartbeat, profound fatigue, pain, perceived cognitive disability such “brain fog”, absent minded, difficulty concentration or confusion, depression, anxiety, and sleep disorder [3]. Lopez-Leon et al. concluded from a systematic review that the five most common symptoms of post COVID-19 were fatigue (58%), headache (44%), attention disorder (27%), hair loss (25%), and dyspnea (24%) [6]. The symptoms of post COVID-19 condition significantly impair health-related quality-of-life (HRQoL) even for those who had mild symptoms and lived active healthy lives before the illness [3]. After the acute phase of COVID-19, Halabchi and colleagues [7] reported that physical rehabilitation and support could be beneficial. Vieira et al. [8] concluded in a systematic review that tele-rehabilitation might improve dyspnea, physical abilities and HRQoL.

So far, no single intervention has been proven to effectively address the complex symptoms of post COVID-19. While the impact of yoga on post COVID-19 specifically has not been studied, research indicates that yoga can positively influence many symptoms commonly linked to the condition. Yoga might be used in rehabilitation of persons with post COVID-19 condition, as the breathing techniques and postures have shown to have a beneficial impact on the lungs and diaphragm [9] and also reduce anxiety and depression [10].

Yoga originates from ancient India and has increased in popular in western society in recent decades and involves different physical movements, holding different postures (called asanas), breathing technique (called pranayama), relaxation and meditation (dhyana) [11]. Yoga is a low-risk and useful practice and there is some evidence for yoga in the management of chronic diseases like chronic heart failure [12], cancer [13], chronic fatigue syndrome [14]. Yoga has also showed improvement in blood pressure [15], reduced anxiety [16], fatigue [14], sleep and cognitive conditions [17] as well as improves the level of HrQoL [18].

Teleyoga is a way of delivering yoga-based rehabilitation in a digital format. The increased use of videoconferencing technologies where the person can participate in training and rehabilitation from home in live sessions and interact with the yoga teacher has potential for increased participation. An RCT which studied people with ankylosing spondylitis showed significant improvement in fatigue, pain and HRQoL after practicing teleyoga twice a week for 3 months [19].

Previous studies showed that teleyoga was safe, feasible and acceptable in persons with long-term illness exemplified as chronic obstructive pulmonary disease and heart failure [20–22]. Teleyoga has also shown promising results regarding improved depression, reducing dyspnea, reducing stress and increasing persons’ well-being [19, 20].

Breathing exercises have been shown to have a positive impact for persons with Covid-19 [23]. People with post COVID-19 condition describe similar symptoms and problems as persons with chronic diseases such as COPD and heart disease. Yoga might therefore be a suitable rehabilitation and treatment for persons with post COVID-19 condition [24]. Digital yoga for rehabilitation has not yet been assessed for the management of Post COVID-19 condition. Therefore, the aim of this study was to explore the feasibility of teleyoga in persons with post COVID-19 condition by assessing adherence, safety and limited efficacy as well as experiences. The research questions of the study were:

1) How is the adherence to teleyoga?

2) Is teleyoga in persons with post COVID-19 conditions safe?

3) What is the limited efficacy and experiences of teleyoga?

## Methods

### Design

A feasibility study evaluating a teleyoga intervention with a pre-post mixed-method design including people with post COVID-19 condition was performed [25]. The method has followed the CLARIFY guidelines for reporting yoga interventions [26].

### Study participants

The participants were recruited from a post COVID-19 condition rehabilitation clinic at a county hospital in south-east of Sweden. Twenty individuals were asked and nine declined, mainly due to extensive fatigue. The study was conducted between May and August 2021. All included patients had had a Covid infection, confirmed by a positive PCR test or antibody test.

### Inclusion criteria and exclusion criteria

Participants were included in the study if they were over the age of 18 years and if they were diagnosed with post COVID-19 condition (defined as symptoms persisted for more than three months after the infection) and in need of rehabilitation. One of the co-authors (MS) was the treating physician at the post-COVID clinic and she invited patients consecutively to participate. Post COVID-19 condition is defined according to WHO as: a laboratory-confirmed SARS-Cov-2 infection with a minimum time of 3 months from date of the positive test. Duration of symptoms for at least 2 months, not explained by an alternative diagnosis and having an impact on everyday functioning [4].

Participants were excluded from the study if they were unable to complete outcome measures and/or to participate in the teleyoga intervention due to different types of very severe limitation based on chart review and screening interviews or an expected survival of less than 6 months.

### The teleyoga intervention

The teleyoga intervention was originally co-designed in collaboration with a patient organisation for heart and lung disease, a certified Mediyoga-instructor, and the IT department at Linköping University and is described elsewhere [21]. The technology used was a tablet with a SIM card and 4G broadband technology, video communication via Zoom and an application with yoga instructions for self-yoga. The application included instructions (text, pictures, and sound files) for yoga positions, breathing and meditation. The medical yoga used was a therapeutic form of Kundalini yoga. The yoga program consists of two standardised programs (Suppl 1) soft light movements, meditation and relaxation and can be done sitting in a chair or on the floor. A session contained 10 min warming up and breathing exercises, 40 min of yoga postures and finally 10 min of meditation. There was technical support available for participants in need. For 7 weeks participants performed a standardised 60-minute teleyoga group session twice a week, led by a certified medical yoga instructor via live video conference-system. The program was not developed for patients with a post-COVID condition but has been tested in patients with long term conditions, such as heart failure, who experience similar symptoms such as fatigue and shortness of breath. In this study, participants were encouraged to lie down and rest during the yoga class and to do less of the yoga exercises if adverse symptom occurred or they became to fatigued. The participants were also encouraged to practice yoga individually using the application with a goal of 50 min per week. For example, one session for at least 10 min a day 5 days/week. After 7 weeks the group yoga ended, but the participants were advised to continue using the application for a minimum of 50 min per week. The yoga instructor was experienced in providing teleyoga to patients with long-term conditions, such as heart failure. Fidelity was monitored and teleyoga was provided by the same instructor and using standardised programmes as described in supplement 1.

### Outcome measures we tested the limited efficacy

#### Adherence

Adherence to the teleyoga intervention was measured by registration of participation by the yoga instructor during the Zoom meetings and by analyzing the log in the application. Telephone calls and email reminders were employed as strategies to enhance adherence. Individual interviews were conducted by telephone at 12 weeks, assessing the reasons for adherence to both the online yoga sessions as the adherence to the application.

#### Safety

Serious adverse events were collected by the yoga instructor and from the medical charts. We constructed a semi-structured interview guide comprising of 16 questions (see Supplementary material 2) and individual interviews were conducted by telephone at 12 weeks to assess adverse events during the intervention. The interviews were recorded via telephone and transcribed verbatim.

#### Limited efficacy

In this feasibility study, we tested the limited efficacy of the intervention, which can be performed with limited statistical power according to Bowen [27]. Therefore, this study examined whether yoga can be delivered online and yield trends in the predicted direction for better outcome in patients with a post COVID-19 condition. Furthermore, we assessed the patients experiences after the teleyoga intervention.

At baseline and 7 weeks clinical measurements were assessed, such as blood pressure, pulse, breathing, physical function using 6 min walk test [28] and self-selected walking speed also called gait speed [29] and cognitive ability measured by Montreal Cognitive Assessment test. Scores equal to or below 26 may indicate cognitive impairment [30]. Socio-demographic data were measured with a self-reported questionnaire and from medical chart, clinical characteristic and comorbidity was collected. Participants also completed questionnaires including questions about socio-demographic data, experience of yoga and technology, HrQoL, mental distress, sleep and exercise motivation. Health-related quality of life was measured using the EuroQol 5-dimensions (EQ-5D) (mobility, self-care, usual activities, pain/discomfort and anxiety/depression). It was the 5-level version that was used (level 1 indicating no problem, level 2 indicating slight problems, level 3 moderate problems, level 4 indicating severe problems and level 5 indicating unable to/extreme problems). EQ-index is derived by applying a formula that attaches values (weights) to each of the levels in each dimension. EQ-VAS range from 0 being the worst health you can imagine to 100 being the best health you can imagine [31]. Symptoms of anxiety and depression were assessed using Hospital Anxiety and Depression Scale, theoretical range from 0 to 21 and cut off for depression and anxiety ≥ 7 [32, 33]. Sleep was measured with the Minimal Insomnia Symptom Scale, (MISS), theoretical range 0–12 cut off ≥ 6 is presence of insomnia [34], and the Exercise Motivation Index [35], theoretical range 0–4. All instruments have validated Swedish versions. Individual interviews were conducted by telephone at 12 weeks to assess the limited efficacy of the yoga intervention based on the experiences of the participants.

### Data analysis

#### Quantitative analysis

IBM SPSS version 28 was used for statistical analysis. Descriptive statistics were used for participant characteristics, safety outcomes and adherence to the teleyoga. Wilcoxon Signed Rank Test was carried out to determine changes from baseline to follow-up after 7 weeks on all outcomes. Two-tailed p-values < 0.05 were considered significant.

#### Qualitative analysis

Qualitative interviews were analysed using inductive content analysis [36]. The interviews were in the first step transcribed verbatim and read narratively. In the second step, two interviews were read by four of the authors (ML, CO, LK, AS) and codes were developed. In step three, the first author identified codes in the remaining interviews. In step four the codes were compared by 4 authors (ML, CO, LK, AS). Discrepancies in coding were discussed until consensus was reached. In step five the first author sorted codes into categories and in step six all the authors had consensus discussions to agree on the final categories. In step six the categories formed headlines, this work was lead by the first author picked up all the sentence-building units under the different subcategories. In step seven the four authors modified and discussed categories.

One of the first authors (ML) was the mediyoga instructor in the intervention and had a long experience of yoga. During the analysis work, the author emphasized awareness of the pre-understanding and pre-conceptions. The analysis was validated through careful discussions with all the other authors. The co-authors had broad experience in clinical research, mixed methods, and content analysis. Three of the authors had clinical experience in COVID-19 care and rehabilitation.

#### Convergent mixed method

The results from the inductive qualitative analysis and the statistical analysis were first completed separately and in the next step the results were combined under each of the 3 research questions in a convergent mixed method approach. We developed a side-by-side joint display represent merging by arraying qualitative and quantitative results next to each other, organised by the research questions [37].

#### Ethical reflections

Eligible participants were informed written and verbally and invited to participate by the staff at the rehabilitation clinic and all included participants signed informed consent. The participants were able to terminate their participation at any time. The study was approved by The Ethical committee of Linköping (Dnr 2017/225-3, Dnr 2020–04891).

## Results

Quantitative and qualitative data are presented both separately and together in a convergent mixed method design addressing the research questions, an overview is shown in Table 1.

**Table 1:**
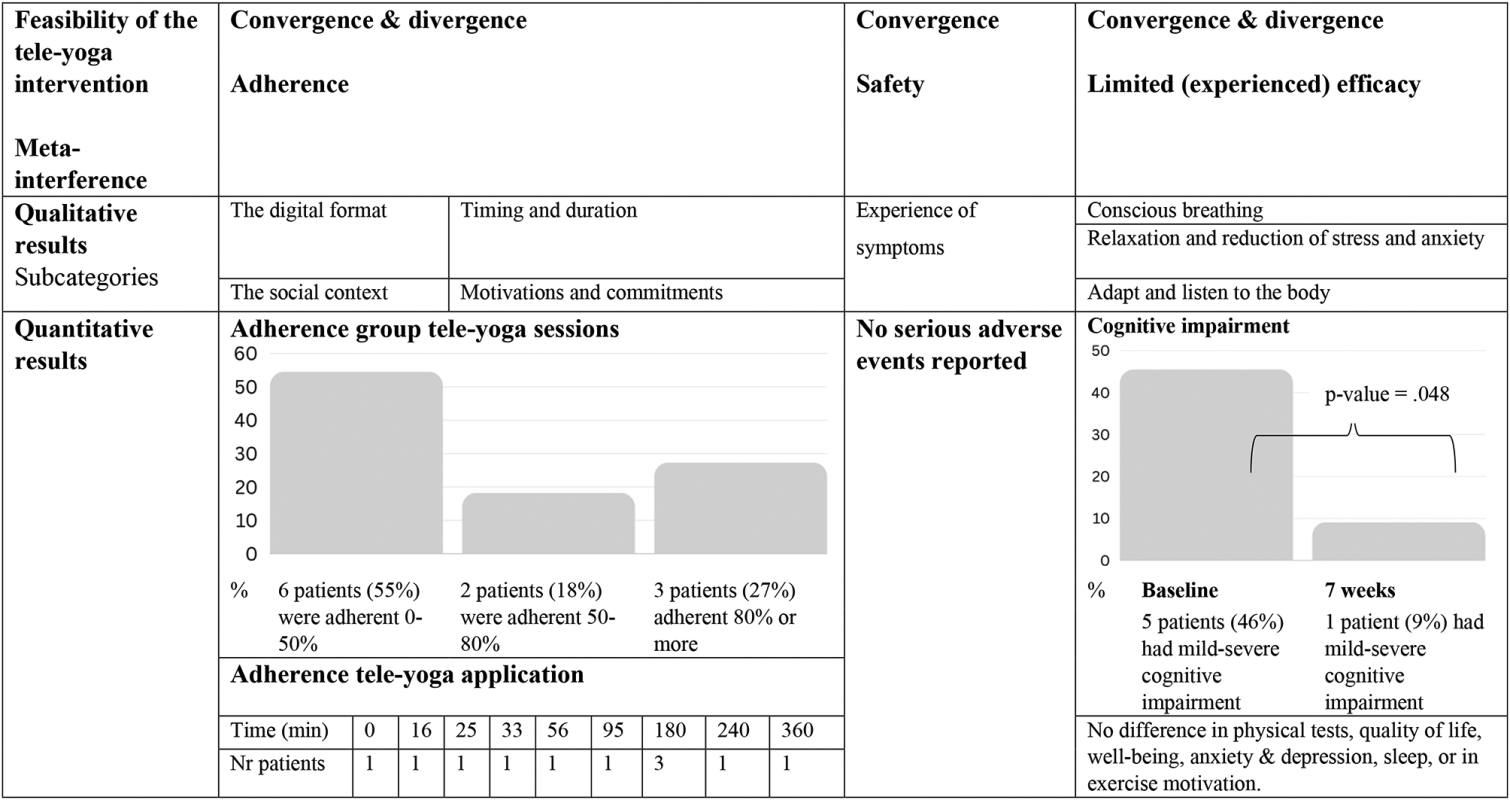
Joint display showing the feasibility of teleyoga in persons with COVID-19 condition

### Participants

Eleven participants finalised baseline and 7 weeks follow up. Mean age of 45 years old and 9 participants were female. All of them suffered from post COVID-19 condition and one of the participants had been hospitalised due to COVID-19. One participant was diagnosed with diabetes; the rest of the participants did not suffer from any comorbidities. Most participants were married (n-=8) with children (*n* = 9) and all participants rated their economy as good. Several participants had previous experiences of yoga. All participants used the internet almost every day and had computer and smartphone skills (see Table 2).

**Table 2:** Demographic and clinical data of 9 persons with post COVID-19 condition

Demographics	*n* (%)
**Female sex**	9 (82%)
**Married**	8 (73%)
**Live with others**	9 (82%)
**Having children**	9 (82%
**Economic situation**	10
Very good	1 (9%)
Good	9 (91%)
**Main occupation**	
Working full-time	5 (50%)
Working part time	2 (20%)
Sick leave	2 (20%)
**Yoga experience**	5 (50%)
**More than 3 h physically active a week**	6 (60%)
**Clinical data**	
6 MWT meters, median (Q1-Q3)	482 (355–540)
Gait speed in meters, median (Q1-Q3)	6 (5–8)
Heart rate, median (Q1-Q3)	80 (73–99)
Blood Systolic mmHg pressure, median (Q1-Q3)	117 (100–131)
Blood Diastolic mmHg pressure, median (Q1-Q3)	80 (80–90)
Respiratory rate/minute, median (Q1-Q3)	14 (11–24)
Cognitive dysfuntion (mean, SD)	6 (11)
Depression, ≥ 7 score on HADs Depression, n (SD)	3 (10)
Anxiety, ≥ 7 on HADs Anxiety, n (SD)	3(10)
EQ-5D Mobility, median (Q1-Q3)	3 (2-3.25)
EQ-5D Selfcare, median (Q1-Q3)	1 (1–2)
EQ-5D Usual activities, median (Q1-Q3)	4(3.4-5)
EQ-5D Pain, median (Q1-Q3)	3 (3–4)
EQ-5D Anxiety, median (Q1-Q3)	2 (1.75–2.25)
EQ_Index median (Q1-Q3)	0.40 (0.25–0.55)
EQ_VAS median (Q1-Q3)	40 (31.25-60)
Total exercise motivation median (Q1-Q3)	2.2 (1.90–2.80)
Sleep quality (MISS) median (Q1-Q3)	5.5 (3.75–8.25)

### Adherence

The participants attended the teleyoga sessions a median of 6 times (range 1–13) during a period of 7 weeks. Three persons (27%) were fully adherent to the teleyoga sessions (attended 80% or more of the teleyoga sessions) and 2 persons (18%) were adherent between 50 and 80% of the teleyoga sessions. The rest were adherent to less than 50% of the teleyoga sessions (6 persons, 55%). The recommendation for yoga application use was 50 min per week for 12 weeks, and a total of 600 min for the whole period. Five out of the 11 participants used the application and with a range between 94 and 360 min during the 12 weeks. None of the participants achieved 80% of the recommended application use.

From the interviews four categories described the adherence of the teleyoga intervention: [1] the digital format; [2] social context; [3] timing and duration; [4] motivations and commitments.

Participants described accessibility as a benefit of the digital format as they did not have to travel to a gym to perform yoga and physical activity could be done at home. It would have been difficult to practice yoga if they had to travel due to physical weakness, fatigue, lack of stamina and energy, and being out of shape after COVID-19. Participants expressed being less comfortable with computers and described difficulties with concentration in front of the screen. The video conferences used during the yoga session created challenges in delivery, such as problems with sound, and the need to charge the tablet. Looking at the experience with the yoga app, participants used the application more in the beginning of the intervention compared to the end. Instead of using the yoga application, participants could choose to do yoga on their own, or use other technology, such as YouTube. Participants expressed that the breathing exercise in the application was the most frequently used feature in the application.

The social context during the group teleyoga sessions with others with the same diagnosis was highly appreciated as they could share experiences with each other. Participants also stated that they would rather have been in a yoga studio for the sessions, as they felt the need to interact with others face-to-face.

Participants expressed that they wanted to have more than two yoga sessions a week, but that they should have been shorter than one hour. This was mostly due to experience of symptoms such as brain fatigue. Participants also found the times of the yoga sessions to be inconvenient (these were in the afternoon) as they could have had more energy if the sessions were given in the morning.

I haven’t really had the energy to do everything at the same time, yoga and work and take care of everything at home.

Participants were satisfied with the yoga instructor and thought it was more motivating to perform yoga with an instructor than on their own. Other reasons to continue were feeling motivated because they experienced mental benefits due to the yoga and they expressed that they were able to do something for themselves and not just take medications. Openness toward complementary medicine and openness to participating in studies was also expressed as a motivator. Participants felt time constraint due to other commitment, for example family commitments, other rehabilitation appointments or holidays.

Analysis of the quantitative and qualitative data showed that half of the participants were adherent to group teleyoga, and the adherence to the yoga application was low. Particpants described that, even if technical challenges could occur, they appreciated the digital format of the intervention and the possibility to be active in a group. There was a challenge in the timing and the duration of the yoga sessions as they felt too fatigued to do yoga. The yoga instructor, experiencing phsyical and mental benefits, openness toward complementary medicine and participating in studies were motivators for be adherent to the teleyoga intervention. Other responsibilities could take time away from teleyoga.

### Safety

There were no serious adverse events reported during the study. The qualitative findings on the safety of the teleyoga showed one category: [1] Experience of symptoms.

The participants experienced various physical symptoms when starting yoga such as headaches, nausea, dizziness, and fatigue, however these experiences decreased over time. They also described low in energy if they tried too hard. The body was vulnerable after the COVID-19 infection and with exercise intolerance and increased symptoms during exercise. Due to heavy headache and brain fatigue, participants choose not to participate in the live streamed yoga sessions and performed yoga on their own instead. Participants had a changed breathing pattern with hyperventilation from covid − 19, and experienced difficulties breathing slowly and deep.

### Limited efficacy

There was a significant improvement in cognition at 7 weeks compared to baseline (*P* = 0.048). At baseline there were 5 participants that were cognitively impaired with a MoCA score < 26. After 7 weeks all but one improved to normal cognition function. At baseline the participant had most problems in visuospatial skills and short-term memory. There was no change in the physical tests, HrQol, nor in depression or anxiety.

The qualitative finding showed three categories on limited efficacy of the teleyoga intervention: [1] conscious breathing; [2] relaxation and reduction of stress and anxiety; [3] adapt and listen to the body.

Participants expressed how they had become more aware of how they breathe. Participants described that after the yoga sessions they felt that they could breathe better and have more control over their breathing. Participants reported that teleyoga improved relaxation and reduced their stress and anxiety.

*I found a lot of peace in yoga*,* I could relax in a way… so the biggest positive I think is just the calm and breathing and in the beginning anyway I had a lot of anxiety. The anxiety was relived very well by it.*

Participants experienced improvement of both body and mind as rewarding and satisfying. The participants expressed that they adapted and listened to their body. Physical symptoms caused the participants to change the way they performed yoga. Having trouble with shoulders or difficulties with having their arms above their head during certain yoga movements made them adapt the movements so that they did not experience pain. They felt encouraged to adjust the movements to their energy levels, like laying down and resting during the yoga sessions or shortening the time of the yoga movements. The participants expressed that they felt motivated to continue as yoga gave them a structure and a feeling of belonging to a group. Another motivation was the desire to recover from their illness and they wanted to try everything which could make this possible.

I feel like I am retired. I want to get back to normal and start working and have a normal life so that everything I - everything they tell me I do - that’s it and yoga was great.

When converting the quantitative and the qualitative results we can state that despite no change in respiratory rate, participants were more aware of their breathing, breathe better and have more control over their breathing. There was also no change in symptoms of anxiety and depression or on the physical test, but participants described how they felt an increase in their wellbeing, a reduction in their stress and anxiety and increase in their physical strength.

## Discussion

The aim of this study was to explore the feasibility of teleyoga in persons with post COVID-19 condition focusing on adherence, safety, limited efficacy and experiences. The main findings were that adherence to the teleyoga sessions was quite low and might be improved with shorter yoga sessions. Online yoga was found to be safe with regard to severe adverse events, but some participants experienced an increase in symptoms. Teleyoga was associated with improved cognition, breathing and relaxation. Teleyoga could be feasible for people post COVID-19, but adaptation and increased flexibility of the yoga-program may be required. As few participants were adherent to the application, the relevance and usefulness of this needs to be further explored.

Our study showed that only one-third of the participants were adherent to 80% or more of the complete teleyoga intervention. Post COVID-19 affected many aspects of the study participants’ lives due to fatigue, brain fog, pain, breathlessness, and many other symptoms. Which is consistent with several other studies which have described symptoms of long-term COVID-19 [38, 39]. The results show that the teleyoga probably needs further adaptation to post COVID-19, especially as many patients experience an increase in symptoms during the yoga practice. Furthermore, the teleyoga should be more flexible on the duration as well as the number of sessions. Participants had a low adherence to the yoga application, and therefore it should be further developed or omitted in future yoga interventions.

Several other barriers to participation may have affected adherence to teleyoga, such as the time of the day when teleyoga sessions were held and if participant were working or not. The intervention was held during the Swedish summer during vacation and adherence to teleyoga intervention may have been better if the intervention was held during another season. Adherence to teleyoga sessions might have been higher with more flexible time of the teleyoga sessions.

The participants in our study described the importance of the social context and group format in the teleyoga sessions. Another study found contradictory results as participants expressed less social interaction in tele-health classes [40].

The participants expressed it as beneficial that they could perform yoga training at home. Rehabilitation in a digital format can potentially increase and facilitate participation for people with post COVID-19 condition and those living far from rehabilitation clinics [41]. However, our study showed a few challenges with the technology, such as concentration difficulties and fatigue while looking at the screen. Using videoconferencing technology at home also provided some challenges. In our study participants had difficulty maintaining privacy and being left alone when having a family. Another study described the same challenges, where participants struggled to create a calm and quiet environment during tele-health classes and could often be interrupted by other family members [40].

Our findings showed no serious adverse advents from the teleyoga intervention, but participants were affected by their post COVID-19 symptoms. For some participants one hour of teleyoga was too long. Some of them experienced worsening symptoms after the yoga exercise. These findings were in line with another study, where they showed physical exertion as the most common cause of worsening of their symptoms [42]. In our study some participants described worsening symptoms in the first two weeks of teleyoga, but experienced less symptoms as time progressed.

After seven weeks of teleyoga there was significant improvement in cognitive function. Participants expressed how they could concentrate and focus better during the day. These results were confirmed in another study showing improved cognition in elderly who participated in physical activities for 20 weeks [41]. Our qualitative results showed a varied experience of teleyoga including an increased awareness of how breathing influenced wellbeing, relief of anxiety, and feelings of belonging to a social context again which was expressed as meaningful. Participants expressed how they felt physically stronger over time and could handle life and cope better after participating in teleyoga. The improvement of both body and mind was experienced as rewarding and satisfying. A literature study showed that aerobic, respiratory, and low intensity exercise is an effective strategy for persons with post COVID-19 condition [43].

After the teleyoga participants experienced their breathing in a new way. They expressed how they felt that they could breathe easier and was more aware and had more control over their breathing. These findings were confirmed by another study showing that tele-rehabilitation increased physical strength and decreased dyspnea in persons suffering from Covid-19 [44]. In a systematic literature review [8], breathing exercise via telerehabilitation for persons with COVID-19 showed an improved physical function.

Tele-rehabilitation have benefits such as saving time and effort by eliminating the need to travel to and from a clinic for appointments [45]. Our study confirmed that it was convenient for participants to be able to perform teleyoga at home. Tele-rehabilitation can be more cost-effective than in-person rehabilitation and can be a viable and effective option for implementation in clinical practice, particularly in cases where in-person visits are not possible or practical [46]. An important issue to discuss is the privacy, confidentiality, and safety of the videoconference platform and the new demands for healthcare professionals.

### Strength and limitation

The results should be interpreted with caution due to the small number of participants, no control group, and the low adherence to teleyoga sessions and app use. Teleyoga sessions used in this study were standardised programs and not developed specifically for COVID-19 nor individually adapted in relation to symptoms or limitations. Further a more gradual increase in the length of the sessions and offering more sessions per week could have been beneficial. Our sample may not have been representative since several participants were well-educated, had a stable economy, were regular internet users and several had tried yoga before.

We used a mixed-method approach by presenting both quantitative and qualitative data thereby providing a more complete and nuanced understanding of the topic being studied [25]. Triangulation involves using multiple sources of data to corroborate or cross-validate findings. This can help to increase the validity and reliability of the research findings [47] Further randomised controlled trials as well as larger sample sizes are desirable for future studies to provide stronger evidence regarding potential post COVID-19 rehabilitation. The first author (ML) was the yoga teacher conducting the sessions. In the qualitative analysis, her extensive background in teaching yoga may have influenced the results. To address this, all authors collaborated extensively throughout various stages of the process and critically evaluated alternative explanations.

## Conclusions

To the best of our knowledge, this is the first study describing teleyoga in persons with post COVID-19 condition. Tele-rehabilitation through therapeutic exercises with long deep breathing and meditation appears to be feasible, safe and may improve cognition. However, the need for larger well-designed studies conducted in a structure and standardised way are needed to ensure the evidence of teleyoga as rehabilitation for persons with post COVID-19. Overall, tele-rehabilitation has the potential to provide significant societal benefits by improving access to rehabilitation services, reducing healthcare costs, and increasing personal convenience and satisfaction.

## Electronic Supplementary Material

Below is the link to the electronic supplementary material.

Supplementary Material 1

Supplementary Material 2

## Data Availability

Due to the nature of the sensitive personal data and study materials, they cannot be made freely available. However, by contacting the corresponding author, procedures for sharing data, analytic methods, and study materials for reproducing the results or replicating the procedure can be arranged following Swedish legislation.
