# Supplementary material for: Feasibility of teleyoga for people with post COVID-19 condition– a mixed method design
Source: BMC Complement Med Ther. 2025 Jan 8;25:6. doi: 10.1186/s12906-024-04735-4 (PMC11715557; doi:10.1186/s12906-024-04735-4)
Supplement: Supplementary file 1 — Supplementary Material 1 [file 12906_2024_4735_MOESM1_ESM.docx]

**Supplementary material 1**

**Medical Yoga programmes**

***Programme-I*** (total of 60 minutes), where participants will learn and practice long-deep breathing. This programme includes two physical postures, spinal flex and Sat Kriya. It includes the heartmeditation and Kirtan kriya finger meditation.

***Programme-II*** (a total of 60 minutes), where participants will practice deep breathing and ‘breath of fire’ exercises. Physical postures will focus on the spine with several exercises that gently turn the back in different directions. Meditation includes breathing meditation with inhalation through the left nostrils and exhalation through the right nostril, and pulse meditation where participants both concentrate on their breathing and feeling their pulse. Relaxation, warm-up, and cool-down will be delivered in both programs.
