# Supplementary material for: Feasibility of teleyoga for people with post COVID-19 condition– a mixed method design
Source: BMC Complement Med Ther. 2025 Jan 8;25:6. doi: 10.1186/s12906-024-04735-4 (PMC11715557; doi:10.1186/s12906-024-04735-4)
Supplement: Supplementary file 2 — Supplementary Material 2 [file 12906_2024_4735_MOESM2_ESM.docx]

**Supplementary material 2**

**Interview guide**

1. Tell me about your experiences with teleyoga.
2. Describe if you have had any positive effects from the yoga?
3. Describe if you have had any negative effects from the yoga (discomfort or worsening of your symptoms)?
4. Tell me about any obstacles you faced when practicing yoga?
5. Were there opportunities for you to adapt the yoga to your abilities and needs?
6. What did you like best about the yoga?
7. What did you like least about the yoga?
8. Did you practice yoga on your own on the days you did not meet with the group?
9. Can you tell me about practicing yoga independently with the help of the application?
10. How often did you use the application?
11. Can you tell me about practicing yoga in a group online (technical aspects and social aspects, etc.)?
12. Did you continue to do yoga after the group ended after 6 weeks? If so, tell me about your experiences of continuing with yoga entirely on your own.
13. Is there anything else you would like to share about what motivated you to do yoga during these three months?
14. If you could choose freely, how would you have liked to practice yoga? For example, when, where, how.
15. What suggestions for improvement do you have regarding teleyoga?
16. Do you have any other thoughts or opinions about the study that you would like to share?
